# Vertical Ionization Potentials and Electron Affinities at the Double-Hybrid Density Functional Level

**DOI:** 10.1021/acs.jctc.3c00363

**Published:** 2023-06-16

**Authors:** Dávid Mester, Mihály Kállay

**Affiliations:** † Department of Physical Chemistry and Materials Science, Faculty of Chemical Technology and Biotechnology, Budapest University of Technology and Economics, Műegyetem rkp. 3., H-1111 Budapest, Hungary; ‡ ELKH-BME Quantum Chemistry Research Group, Műegyetem rkp. 3., H-1111 Budapest, Hungary; ¶ MTA-BME Lendület Quantum Chemistry Research Group, Műegyetem rkp. 3., H-1111 Budapest, Hungary

## Abstract

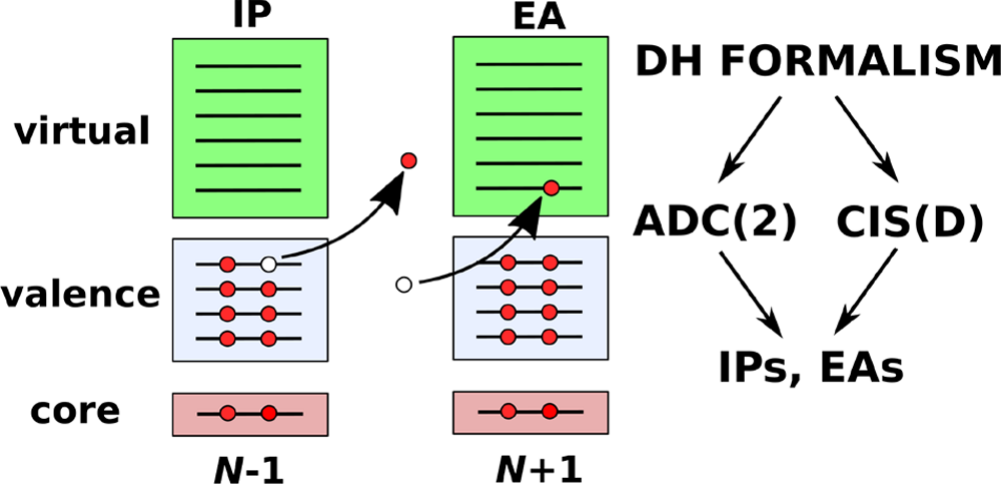

The double-hybrid (DH) time-dependent density functional theory is extended to vertical ionization potentials (VIPs) and electron affinities (VEAs). Utilizing the density fitting approximation, efficient implementations are presented for the genuine DH ansatz relying on the perturbative second-order correction, while an iterative analogue is also elaborated using our second-order algebraic-diagrammatic construction [ADC(2)]-based DH approach. The favorable computational requirements of the present schemes are discussed in detail. The performance of the recently proposed spin-component-scaled and spin-opposite-scaled (SOS) range-separated (RS) and long-range corrected (LC) DH functionals is comprehensively assessed, while popular hybrid and global DH approaches are also discussed. For the benchmark calculations, up-to-date test sets are selected with high-level coupled-cluster references. Our results show that the ADC(2)-based SOS-RS-PBE-P86 approach is the most accurate and robust functional. This method consistently outperforms the excellent SOS-ADC(2) approach for VIPs, although the results are somewhat less satisfactory for VEAs. Among the genuine DH functionals, the SOS-ωPBEPP86 approach is also recommended for describing ionization processes, but its performance is even less reliable for electron-attached states. In addition, surprisingly good results are attained by the LC hybrid ωB97X-D functional, where the corresponding occupied (unoccupied) orbital energies are retrieved as VIPs (VEAs) within the present formalism.

## 1 Introduction

Vertical ionization potentials (VIPs) and electron affinities (VEAs) are crucial parameters that are used to characterize the electronic structure of molecular systems. The accurate prediction of VIPs, for example, can provide insights into the properties of semiconducting materials used in solar cell devices. Therefore, understanding the relationship between VIPs and photovoltaic efficiency is of great importance in the development of next-generation solar cells.^1−3^ Furthermore, ionizing radiations can cause damage to DNA, with secondary electron attachment resulting in various forms of DNA degradation, making it important to understand the role of electron-attached states in genetics, biochemistry, and health sciences.^4−10^ The reliable prediction of these parameters for extended molecular systems is still a challenging task that requires accurate and efficient theoretical methods.

To obtain the total energy of a given system, the most commonly used approaches are based on the cost-effective density functional theory (DFT).^11^ Within DFT, the highest occupied Kohn–Sham (KS) orbital energy of an *n*-electron system can also be interpreted as the negative of the exact ionization potential;^12−14^ however, these values are often underestimated in comparison with the experimental results.^12,15^ Consequently, the use of the more advanced wave function-based methods is desired, but a good alternative could also be offered by the efficient double-hybrid (DH) DFT approaches, which combine KS-DFT with second-order wave function approximations.^16−21^

Concerning the wave function methods, the equation-of-motion coupled-cluster (EOM-CC) formalism^22−26^ is a popular computational tool for calculating electronic excited states of molecules. It is based on the similarity transformation of the electronic Hamiltonian, and the resulting effective operator is diagonalized to produce final state wave functions and energies. One of the most significant advantages of the EOM-CC approach is its ability to systematically include electron correlation effects in the calculations, enabling one to achieve arbitrary accuracy within the hierarchical expansion. However, as the methods become more advanced, they also become more expensive. The EOM-CC formalism can describe a qualitatively different set of final states for an *n*-electron reference system. For instance, ionization processes can be studied by diagonalizing the corresponding operator in a basis of determinants containing *n* – 1 electrons,^27−31^ while electron-attached states correspond to the diagonal representation in the *n* + 1 particle space.^32,33^

Another promising approach in the field is the algebraic-diagrammatic construction (ADC) formalism.^34^ This method is based on the diagrammatic perturbation expansion of the polarization propagator and the Møller–Plesset (MP) partitioning of the Hamiltonian. Similar to the EOM-CC approach, the ADC formalism also permits the indirect inclusion of orbital relaxation effects through couplings to higher-excited configurations. Originally, for VIP and VEA calculations, the ADC methods were elaborated based on the perturbative self-energy expansion of the Dyson equation.^35^ However, this led to computationally inefficient schemes as the electron attachment and detachment parts of the propagator are coupled. To address this issue, non-Dyson ADC schemes were later proposed,^36,37^ which allow for independent calculations of VIPs and VEAs. The effective implementation and comprehensive investigation of the ADC methods is still an active research field.^38−44^

Further Dyson propagator methods must also be mentioned,^45^ which are not strictly related to the ADC formalism. These methods are based on an approximation of the self-energy matrix, where the diagonal elements of the corresponding matrix represent a correlated, energy-dependent potential which is felt by the electrons assigned to canonical molecular orbitals (MOs). In the simplest approaches, the mixing of canonical orbitals in the Feynman–Dyson amplitudes is prevented by neglecting the non-diagonal elements of the self-energy matrix.^46,47^ Later, several renormalized methods with more flexible non-diagonal self-energies have also been developed.^47−50^ The performance of such methods has been demonstrated in excellent studies.^47,51^

In the previously mentioned methods, VIPs and VEAs are calculated directly in a single calculation. The so-called energy difference or Δ-approaches, however, follow a significantly different scheme, where the corresponding quantities are obtained as the difference between the total energy of the neutral and charged system computed in two separate calculations using the ground-state approximation. Depending on the applied method, several different approaches can be defined, such as the self-consistent field (ΔSCF),^52−54^ ΔMP,^52^ and ΔCC^55−57^ methods. The major drawback of these approaches is that the optimization of the non-Aufbau determinant can easily lead to variational collapse. Moreover, the resulting excited states are not orthogonal to the ground state, which means that the evaluation of transition matrix elements is highly problematic. In addition, higher-order Δ-approaches often require additional calculations to help the selection of the reference determinant for a desired state. In order to avoid misunderstandings, we would like to note that other ΔMP methods have also been developed.^58,59^ In these cases, the VIPs and VEAs are defined as the difference in the MP correlation energy between the natural and charged systems, which both are described with the orbitals and orbitals energies obtained for the *n*-electron system.

Concerning DH DFT, so far the most widespread option to compute VIPs and VEAs has been to employ this simple but less favorable approach. The performance of DHs for VIPs and VEAs utilizing the Δ-approach is well-documented in the literature. For the interested reader, we recommend the diverse GMTKN55 benchmark study,^60^ where the performance of several popular DHs is tested for well-established benchmark compilations using theoretically back-corrected experimental reference values^61,62^ and is also assessed against numerous pure and hybrid functionals. At the same time, we would like to note that numerous developments have taken place in the field recently. One of the most notable attempts is the orbital-optimized DH scheme using the optimized-effective-potential method,^63^ for which a range-separated version was also elaborated.^64^ In these cases, the VIPs and VEAs are obtained by calculating derivatives of the total energy by finite differences using fractional electron numbers.^65−67^ Another direct method has also been developed^68^ which is based on a more general extension of the second-order energy derivative with respect to occupation numbers,^69^ while the combination of the extended Koopmans’ theorem and the adiabatic connection formalism is also noteworthy.^70^

In this paper, a somewhat different route is followed, and the time-dependent DFT (TDDFT) formalism is extended to directly calculate VIPs and VEAs within the DH theory. We first provide a brief overview of the time-dependent approaches used, including a discussion of the genuine formalism of DH TDDFT theory and a more advanced iterative scheme. Thereafter, the corresponding working equations and efficient implementations for VIPs and VEAs are presented in detail. As we will see, the presented perturbative approach can be considered as a simplified version of the scheme introduced in ref (63). Finally, state-of-the-art and popular functionals are tested on benchmark compilations, and the performances of the methods are comprehensively assessed.

## 2 Theoretical Overview

### 2.1 Double-Hybrid Density Functional Theory for Excitations

The DH theory surpasses popular hybrid TDDFT calculations by incorporating the impact of double excitations. Similar to ground-state calculations,^16^ excitation energies within the genuine formalism^71^ are obtained in a two-step manner. First, in the most common and convenient approach, a Hermitian eigenvalue equation relying on the Tamm–Dancoff approximation (TDA)^72^ is solved as
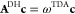
1where **A**^DH^ denotes the corresponding DH Jacobian, **c** is the singles excitation vector, and ω^TDA^ is the TDA excitation energy. Using the spatial-orbital representation, the elements of the Jacobian are defined by
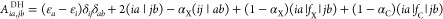
2where *i*, *j*, ... (*a*, *b*, ...) denote occupied (unoccupied) molecular orbitals, and ε_*a*_ and ε_*i*_ are the corresponding orbital energies. (*ia*|*jb*) is a two-electron repulsion integral in Mulliken’s convention, whereas (*ia*|*f*_X_|*jb*) and (*ia*|*f*_C_|*jb*) are the integrals of the exchange and correlation kernels, respectively. The above expression contains two adjustable parameters: the ratio of the Hartree–Fock (HF) and DFT contributions to the exchange energy is handled by α_X_, while the DFT correlation part is scaled by 1 – α_C_. The excitation energies obtained in this way have just hybrid quality. With the solution of eq 1 at hand, in the second step, the second-order correction is calculated perturbatively relying on the configuration interaction singles (CIS)^73^ with perturbative doubles [CIS(D)]^74^ approach. Accordingly, the improved excitation energy at the DH level is obtained as
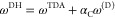
3where ω^(D)^ is the perturbative correction. The thorough theoretical background of genuine excited-state DH approaches has been presented in excellent papers,^71,75−77^ while the efficient calculation of the second-order terms invoking the density fitting (DF) approximation has been detailed in our previous work.^78^

Nowadays, one of the most prominent second-order approaches is the second-order ADC [ADC(2)] method^79,80^ as it offers an appropriate compromise between accuracy and computational cost.^81^ In practice, a nonlinear eigenvalue equation is solved iteratively as
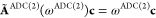
4where  is the so-called effective ADC(2) Jacobian and ω^ADC(2)^ denotes the ADC(2) excitation energy. The corresponding Jacobian can be split into two parts as
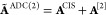
5where **A**^CIS^ is the CIS Jacobian, and all of the terms including second-order contributions are collected into matrix **A**^[2]^.

Similar to CIS(D), an ADC(2)-based DH analogue can also be defined.^82^ In the former case, the CIS excitation energy and singles excitation vector are replaced by the quantities obtained by eq 1, and the second-order correction is scaled by an empirical factor and added to the final excitation energy. For the ADC(2)-based approach, the ADC(2) Jacobian is modified in a very similar way. That is, the CIS Jacobian in eq 5 is replaced by the DH Jacobian defined by eq 2, while the second-order terms are scaled by an empirical factor:
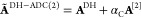
6In contrast to the genuine formalism, where the doubles correction is added *a posteriori* to the TDA excitation energy, these excitations are treated iteratively in this ansatz. Thus, an improvement in the calculated excitation energies is expected. Additionally, the transition properties, such as oscillator strengths, can be calculated at a higher level using the presented approach. Needless to say, the computational cost also increases since the ADC(2)-based approach is iterative.

In recent years, significant developments have been made in the DH theory. First, the spin-scaling techniques have been extended to excited-state calculations as well.^76^ In this case, the opposite-spin and same-spin contributions to the second-order correction are scaled separately,^83−88^ which enables higher flexibility of the energy functional. It is also noteworthy that the computational scaling of the spin-opposite-scaled (SOS) variant, where the same-spin contributions are completely neglected, can be reduced to *N*^4^ invoking the DF approximation for the electron-repulsion integrals and Laplace transform-based techniques,^88^ whereas the scaling of the original and spin-component-scaled (SCS) variants are *N*^5^, where *N* is a measure of the system size.

Later, to remedy the wrong long-range behavior of global exchange-correlation (XC) functionals, long-range corrected (LC) and range-separated (RS) DH functionals have been introduced.^78,89^ In these approaches, the Coulomb operator is split into long- and short-range components using a range-separation parameter. For the RS-DH approaches,^78^ both the exchange and correlation contributions are separated, while only the former one is split for LC DHs.^89^ These approaches can arbitrarily be combined with spin-scaling techniques,^77,90^ whereas their generalization to the ADC(2)-based formalism is also fairly straightforward.^91^

In the following sections, we discuss the extension of DH TDDFT theory to ionized and electron-attached states. Since a TDDFT calculation with a pure or hybrid functional just returns (the negative of) the corresponding orbital energies, this practically requires the generalization of the CIS(D) and ADC(2) methods to VIP and VEA calculations. We also present the working equations for an efficient, DF-based implementation. The adaptation of the approaches for spin-scaled and range-separated functionals is not discussed in detail; the derivation of the corresponding equations is straightforward based on our previous publications.^78,90,91^

### 2.2 VIP and VEA Calculations for CIS(D)

We start our proposition with the evaluation of the second-order correction for the ground state, which is required for the subsequent discussion of the excited-state corrections. Assuming a closed-shell system and spatial orbitals, the second-order MP (MP2) correlation energy is defined by
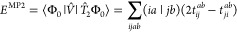
7where the MP2 doubles amplitudes, *t*_*ij*_^*ab*^, can be obtained by acting the  operator on the HF reference determinant, Φ_0_:
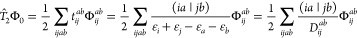
8where Φ_*ij*_^*ab*^ is the corresponding double substitution, and the orbital energy differences *D*_*ij*_^*ab*^ are constructed as ε_*i*_ + ε_*j*_ – ε_*a*_ – ε_*b*_. Using the DF approximation, the four-center quantities can be recast as
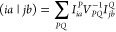
9where *P* and *Q* stand for the elements of the auxiliary basis, whereas *I*_*ia*_^*P*^ and *V*_*PQ*_ are three- and two-center Coulomb integrals, respectively, and *V*_*PQ*_^–1^ is a simplified notation for the corresponding element of the inverse of the two-center Coulomb integral matrix. Usually, the matrix **K** with elements *K*_*ia*,*jb*_ = (*ia*|*jb*) is factorized as **K** = **IV**^–1/2^**V**^–1/2^**I**^T^ = **JJ**^T^. Using the latter notation, the MP2 correlation energy can be expressed in the following simple form:
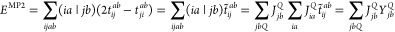
10where the intermediate *Y*_*ia*_^*Q*^ is defined by the contraction of the three-center integrals and the antisymmetrized amplitudes.

For the extension of the genuine DH TDDFT approach to electron-attached/detached states, the generalization of CIS(D) to such states is required. First, we note that the CIS method^73^ can formally be generalized to calculate VIPs and VEAs. Concerning the former, using a generic ionization operator, , the ionized state can be expanded as a linear combination of singly ionized determinants: . Projecting onto the subspace of ionized determinants, the following eigenvalue equations are obtained:
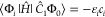
11Here, the eigenvalues of this equation correspond to the negative occupied orbital energies as VIPs, while the eigenvectors are orthogonal unit vectors. The second-order (D) correction for the ionization of the *k̅*th orbital can be interpreted as a CIS(D)^74^ calculation using the corresponding ionized determinant:
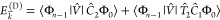
12where the final state can be obtained as
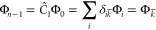
13while the action of the double excitation operator on the reference yields
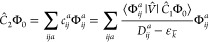
14

As the disconnected terms in the second contribution to eq 12 yield the MP2 energy, the final perturbative correction to the VIP is given as
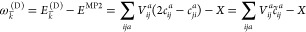
15where the elements of the -related intermediate *V*_*ij*_^*a*^ can be expressed as
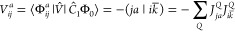
16while the remaining -related contribution of eq 12 is simply calculated as
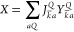
17We note that these equations retrieve the second-order self-energies in the diagonal and frequency-independent approximations, which correspond to the ΔMP2 method.^58,59,92,93^ Accordingly, a similar DH ansatz is recovered from the optimized-effective-potential method of Toulouse and co-workers^63,64^ if the variation of the orbitals and orbital energies in the MP2 correlation energy is neglected when taking the derivative of the total energy with respect to *n*.

As can be seen, in contrast to the standard excited-state CIS(D) equations, the most demanding operations are proportional to *N*^4^. Since one of the occupied indices on the right-hand side of eq 16 is restricted to the ionized orbital, the computation of *V*_*ij*_^*a*^ scales as *N*_occ_^2^*N*_unocc_*N*_aux_, where *N*_occ_, *N*_unocc_, and *N*_aux_ are the number of occupied, unoccupied, and auxiliary orbitals, respectively. The rate-determining step of eq 17 is the evaluation of intermediate **Y**, which also requires only a fourth-power scaling operation due to the restriction. This means that the cost of the perturbative second-order correction is comparable to a single iteration in a ground-state HF calculations. In addition, the unoccupied–unoccupied block of the three-center integrals is not required for the calculations, which is not true for standard CIS(D) calculations.

Similar equations are obtained for the electron-attached states. In this case, for the first-order equations, the energy of the unoccupied orbitals is retrieved as VEAs. The second-order (D) correction for the *c̅*th orbital is calculated as
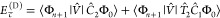
18where the wave function of the *n* + 1-electron state is expressed as
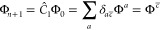
19while the corresponding  operator generates the higher-order excitations:
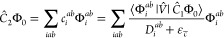
20

The final (D) correction to the VEA is obtained as
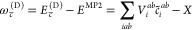
21In this case, the intermediate *V*_*i*_^*ab*^ can be written in the
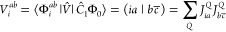
22form, while the intermediate *X* can be calculated as
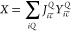
23Again, the rate-determining steps of the above scheme, that is the calculation of *V*_*i*_^*ab*^ and , scale as *N*^4^.

### 2.3 VIP and VEA Calculations for ADC(2)

For the ADC(2) part of the DH calculation, we propose to employ the non-Dyson ADC approach of Schirmer and co-workers.^36,37^ The working equations for VIPs within the non-Dyson ADC schemes up to third order have been previously presented in the literature.^38^ However, an effective implementation relying on the DF approximation has not yet been published. Additionally, even though the derivation of the equations is fairly straightforward, the expressions for VEAs have not been presented. To remedy this, the most important expressions are collected herein.

For VIP calculations, the elements of the vector  are given as
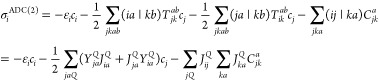
24The resulting terms are very similar to those obtained for CIS(D). However, as the solutions of the corresponding eigenvalue equation are not unit vectors, the final state is properly expanded as Φ_*n*–1_ = *∑*_*i*_*c*_*i*_Φ_*i*_ ≠ *∑*_*i*_δ_*ij*_Φ_*i*_. Accordingly, the elements of intermediate *V*_*ij*_^*a*^ can be expressed as
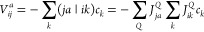
25In addition, the leading complexity of the expressions is somewhat higher compared to that obtained for CIS(D). That is, the rate-determining step of the above procedure is also the evaluation of intermediate **Y**, but the restriction regarding the ionized orbital cannot be applied in this case. This implies that the most expensive step is proportional to *N*^5^; however, it has to be carried out only once, regardless of the number of ionized states. The iterative procedure still scales as *N*^4^ since the intermediate in parentheses can be evaluated before it. In addition, the demanding *J*_*ab*_^*Q*^-type integrals are not required for the calculations.

For VEAs, the elements of the Jacobi matrix transformation read explicitly as
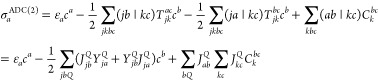
26where *V*_*i*_^*ab*^ is calculated as
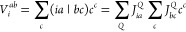
27As can be seen, similar findings can be stated regarding the scaling of the procedure as those obtained for VIPs.

The anticipated benefit of the ADC(2)-based formalism over the CIS(D)-based one is twofold. First, as the singles coefficients are relaxed during the iterative procedure, an improvement in the calculated VIPs and VEAs is expected. Second, other quantities, such as Dyson orbitals and pole strengths,^94−96^ can be calculated at a higher level taking into account the effect of double excitations. As the perturbative (D) correction is only an energy correction for the CIS(D)-based approaches, the aforementioned quantities have only hybrid quality. This implies that pole strengths are always equal to unity in these cases. The calculation of pole strengths for ADC(2)-based DH approaches is fairly straightforward. Similar modifications for transition density matrices have already been discussed in ref (82). That is, the first-order contributions to the spectroscopic amplitude vector are kept unchanged, while all the second-order contributions are scaled by α_C_. The working equations to calculate pole strengths for ADC(2) can be found in ref (38).

## 3 Computational Details

### 3.1 Calculation of the Numerical Results

In this study, the Mrcc suite of quantum chemical programs^97^ was used to calculate VIPs and VEAs, and TDDFT calculations were carried out using TDA. To tackle the corresponding ionized and electron-attached states, a modified Davidson algorithm using the root-following technique^98^ was adopted. Dunning’s correlation consistent basis sets (cc-pV*X*Z, where *X* = D and T)^99,100^ and their diffuse function augmented variants (aug-cc-pV*X*Z)^101^ were employed for the calculations, and the DF approximation was utilized for both the ground and attached/detached states. For this purpose, the corresponding auxiliary bases of Weigend and co-workers^102−104^ were employed. The frozen core approximation was employed in a similar manner to the original benchmark studies (see Sect. 3.2) in the post-KS/HF steps. The convergence threshold for the energies was set to 10^–6^ E_*h*_, while the default adaptive integration grid of the Mrcc package was used for the XC contributions.^105^

For the calculations, the exchange and correlation functionals of Perdew, Burke, and Ernzerhof (PBE),^106^ Becke’s 1988 exchange functional (B88),^107^ the correlation functional of Lee, Yang, and Parr (LYP),^108^ Perdew’s 1986 correlation functional (P86),^109^ and Becke’s 1997 exchange and correlation functionals (B97)^110^ were used. The built-in functionals of the Mrcc package were employed in all cases, except for the LC hybrid and LC-DH functionals, where the locally modified version of the Libxc library^111,112^ was utilized. The attributes of the assessed DH functionals are collected in Table 1.

**Table 1:** Functionals Assessed in the Benchmark Calculations^a^

Functional	Exchange	Correlation	Class	Number of parameters	References
SCS-RS-PBE-P86	PBE	P86	RS DH	4	(90)
SOS-RS-PBE-P86	PBE	P86	RS DH	3	(90)
SCS-ωPBEPP86	PBE	P86	LC DH	7	(77)
SOS-ωPBEPP86	PBE	P86	LC DH	5	(77)
DSD-PBEP86	PBE	P86	global DH	4	(113)
PBE0-2	PBE	PBE	global DH	2	(114)
SOS-PBE0-2	PBE	PBE	global DH	3	(115)
PBE-QIDH	PBE	PBE	global DH	2	(116)
B2GPPLYP	B88	LYP	global DH	2	(117)
ωB97X-D	B97	B97	LC hybrid	18	(118)
CAM-B3LYP	B88	LYP	LC hybrid	3	(119)
PBE0	PBE	PBE	global hybrid	1	(120)

a Both the CIS(D)- and ADC(2)-based approaches are discussed for all the DH functionals, except for SOS/SCS-ωPBEPP86. For more details regarding the ADC(2)-based formalism, see refs (82) and (91).

To gain some insight into the performance of the corresponding methods for valence excitations, we recommend comprehensive benchmark studies^77,90,91,121^ for the interested readers.

The errors utilized for the evaluation of the VIPs and VEAs are calculated by subtracting the reference values from the computed ones. The statistical error measures presented in the figures are the mean absolute errors (MAEs), standard deviations (SDs), and maximum absolute errors (MAXs). All the computed raw energies are available in the Supporting Information (SI). In addition, the mean errors, deviations spans, and root-mean-square errors are also included.

### 3.2 The Benchmark Sets

Three different benchmark sets were selected from the literature to assess the performance of the methods, and currently, these compilations are regarded as the most comprehensive ones for VIP and VEA calculations. First, we discuss the benchmark compilation of Sherrill et al.,^122^ which contains 24 medium-sized organic acceptor molecules. This test set incorporates the first VIPs and VEAs of the corresponding systems. References obtained at the CCSD(T)/aug-cc-pVDZ level are used in this study, invoking the frozen core approximation.

Thereafter, the test set originally compiled by Bartlett and co-workers is analyzed.^123^ Since this compilation was later simplified by Ortiz et al.,^47^ it is hereafter referred to as the BO benchmark set. The final set includes 170 valence ionized states of 33 small molecules using CCSDT/cc-pVTZ results as the reference. As can be seen, this test set contains several VIPs associated also with lower-lying valence orbitals. In contrast to the statistics presented in ref (124), all the VIPs are considered in this study. Similar to the original work,^123^ the core electrons were correlated during the calculations.

Finally, the performance of the functionals for biochemically relevant systems is assessed. A benchmark compilation containing nucleobases was proposed by Śmiga and co-workers.^125^ Recently, high-level VIPs and VEAs were computed by Tajti et al.^124^ using a composite reference at the complete basis set (CBS) limit. As the CBS extrapolation is not trivial for DH-TDDFT calculations, in this study, values obtained at the CCSD(T)(a)*/cc-pVDZ level are used as the references. The benchmark set incorporates six electron-attached/detached states of five different molecules.

## 4 Results and Discussion

In this section, the performance of the considered functionals is assessed. The results are discussed mainly from two aspects. First, the benefits of the ADC(2)-based formalism are compared with the CIS(D)-based one. Second, the effects of the DH formalism are examined in comparison with the corresponding wave function-based approaches. The importance of the spin-scaling techniques has already been emphasized by Tajti et al.^124^ Accordingly, we do not discuss the outcomes from this aspect, especially for the wave function-based methods.

### 4.1 The Acceptor Test Set

**Figure 1:**
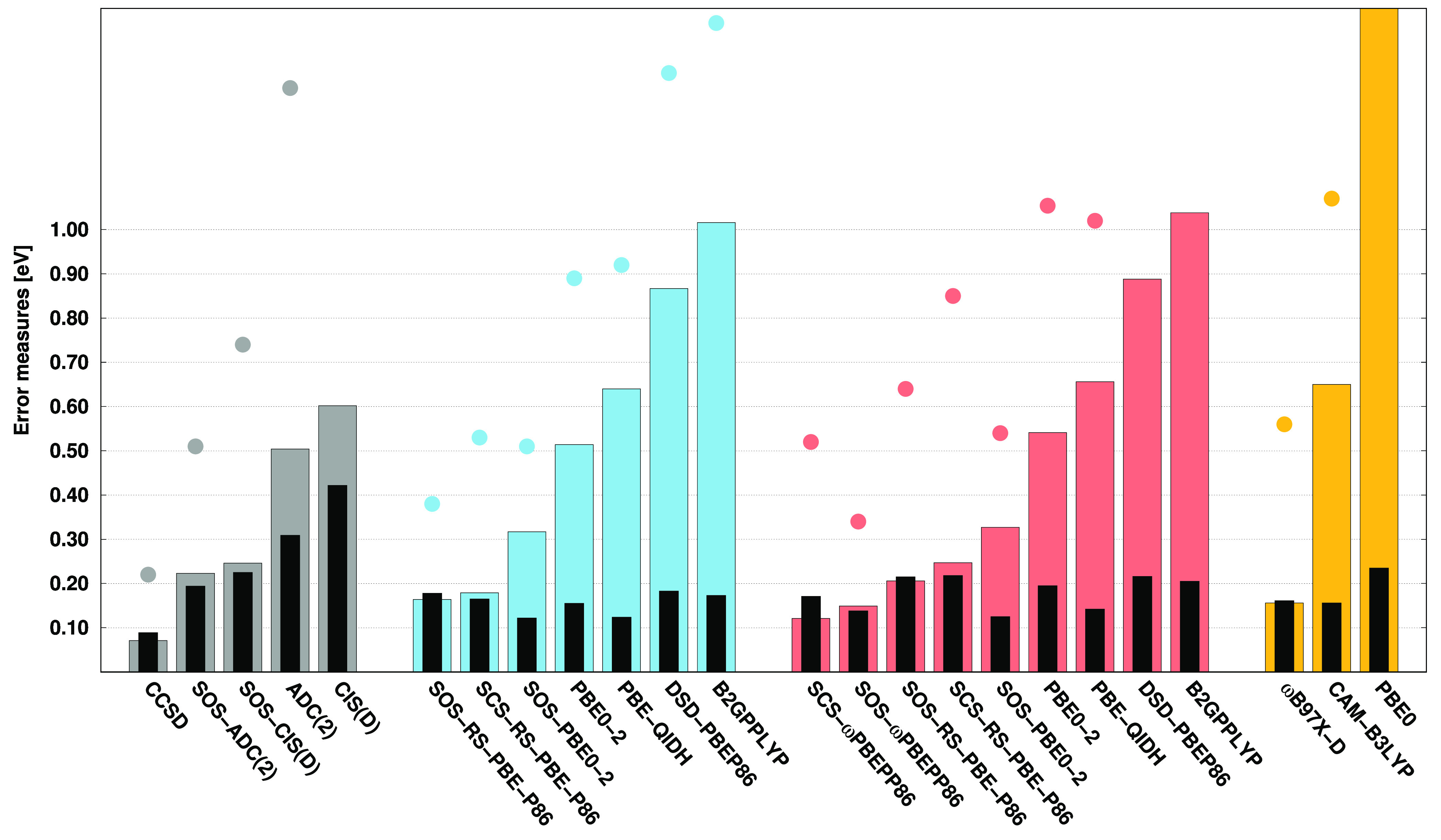
Error measures for the VIPs of the acceptor test set^122^ using the aug-cc-pVDZ basis set with the corresponding auxiliary bases. The MAEs (SDs) are visualized by colored (black) bars, while MAXs are presented by dots. For the sake of clarity, some outliers are omitted. The wave function (first group), ADC(2)-based DH (second group), CIS(D)-based DH (third group), and hybrid methods (fourth group) are presented in gray, blue, red, and orange, respectively. The CCSD values were taken from ref (124).

The acceptor test set of Sherrill and co-workers^122^ is discussed first. The error measures corresponding to the VIPs are visualized in Figure 1. Upon examining the MAEs, one can see that the highest accuracy is achieved by CCSD, with a MAE of 0.07 eV. These error measures for the SOS variant of ADC(2) and CIS(D), which are around 0.22 and 0.25 eV, respectively, are also acceptable. Among the ADC(2)-based DH methods, the best performances are attained by the spin-scaled RS-DH functionals. The MAEs for the SOS and SCS variants are 0.16 and 0.18 eV, respectively, while for SOS-PBE0-2, it is still around 0.30 eV. However, for the remaining functionals, the error exceeds 0.50 eV. It is somewhat surprising for DSD-PBEP86, which also contains spin-scaling parameters. Regarding the CIS(D)-based DH approaches, it can be concluded that the ADC(2)-based counterpart consistently outperforms the genuine variants. Nevertheless, SCS/SOS-ωPBEPP86 yields excellent results with MAEs of 0.12 and 0.15 eV. In addition, ωB97X-D is also outstanding with a MAE of 0.16 eV, while the error is significantly larger for CAM-B3LYP. A somewhat more balanced performance can be observed for the ADC(2)- and CIS(D)-based variants if the SDs and MAXs are considered. In this case, the SDs for the ADC(2)-based approaches range between 0.12 and 0.18 eV, while for the CIS(D)-based ones, they are between 0.14 and 0.22 eV. The MAXs for the most reliable DH methods are approximately 0.50 eV. These results can be achieved by ωB97X-D as well. In general, we can conclude that the error measures for the best functionals are lower in comparison with the corresponding wave function-based counterpart.

**Figure 2:**
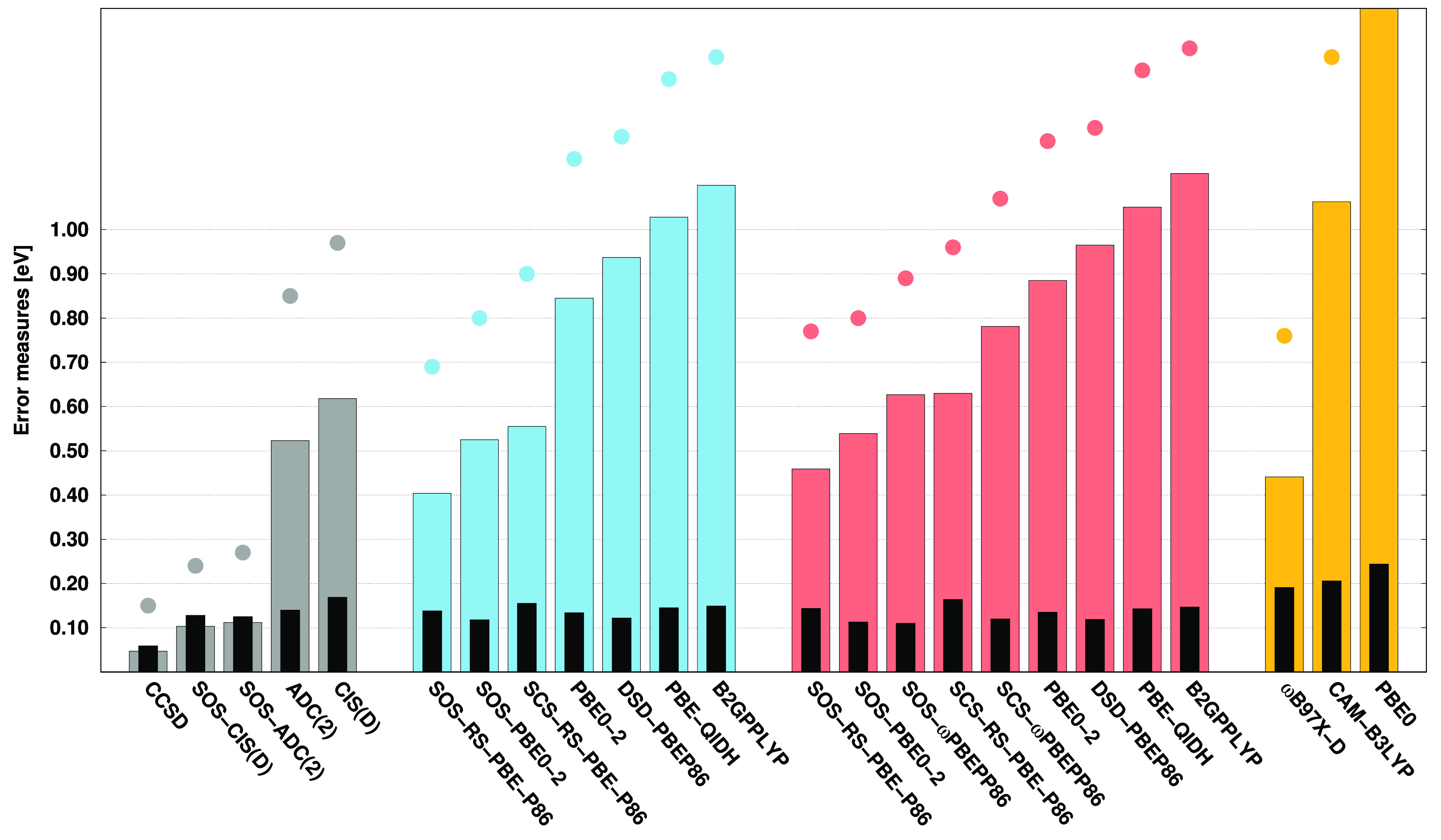
Error measures for the VEAs of the acceptor test set^122^ using the aug-cc-pVDZ basis set with the corresponding auxiliary bases. See the caption of Figure 1 for further details.

Continuing the previous study, the error measures regarding the VEAs are presented in Figure 2. As can be seen, it is difficult to compete with the CCSD, SOS-CIS(D), and SOS-ADC(2) approaches. The lowest MAE, precisely 0.05 eV, is attained by CCSD, while the errors are still around 0.10 eV for SOS-CIS(D) and SOS-ADC(2). Among the ADC(2)- and CIS(D)-based DH functionals, the best performances are provided by the SOS-RS-PBE-P86 functionals. Unfortunately, in these cases, the MAEs are 0.40 and 0.46 eV, respectively. Nevertheless, these values are somewhat lower than those obtained for the standard ADC(2) and CIS(D) methods. This is also true for SOS-PBE0-2, while the remaining DH functionals have significantly higher MAEs. Acceptable performance is achieved by ωB97X-D, with a MAE of 0.44 eV. Inspecting the SDs, a somewhat more balanced picture can be obtained. The precision of CCSD is still outstanding with an SD of 0.06 eV; however, the other methods are very close to each other. That is, the SDs are 0.14 and 0.17 eV for ADC(2) and CIS(D), respectively, while they are around 0.13 eV for the SOS variants. The deviations do not exceed 0.15 eV for most of the DH functionals, while some of the best performers, such as the SOS-PBE0-2 functionals, have even lower SDs compared to the SOS-ADC(2) approach. A bit larger deviation, precisely 0.19 eV, is yielded by ωB97X-D. If the MAXs are considered, the performances are very similar to those obtained for the MAEs. Accordingly, the lowest MAXs, being 0.15, 0.24, and 0.27 eV, are achieved by CCSD, SOS-CIS(D), and SOS-ADC(2), respectively. These excellent results cannot be approached by any other methods. That is, the same measures are 0.85 and 0.97 eV for the standard ADC(2) and CIS(D) approaches, respectively. Among the DH functionals, the best results are provided by the ADC(2)- and CIS(D)-based SOS-RS-PBE-P86, with MAXs of 0.69 and 0.77 eV, respectively, while it is 0.76 eV for ωB97X-D.

### 4.2 The Bartlett/Ortiz Test Set

**Figure 3:**
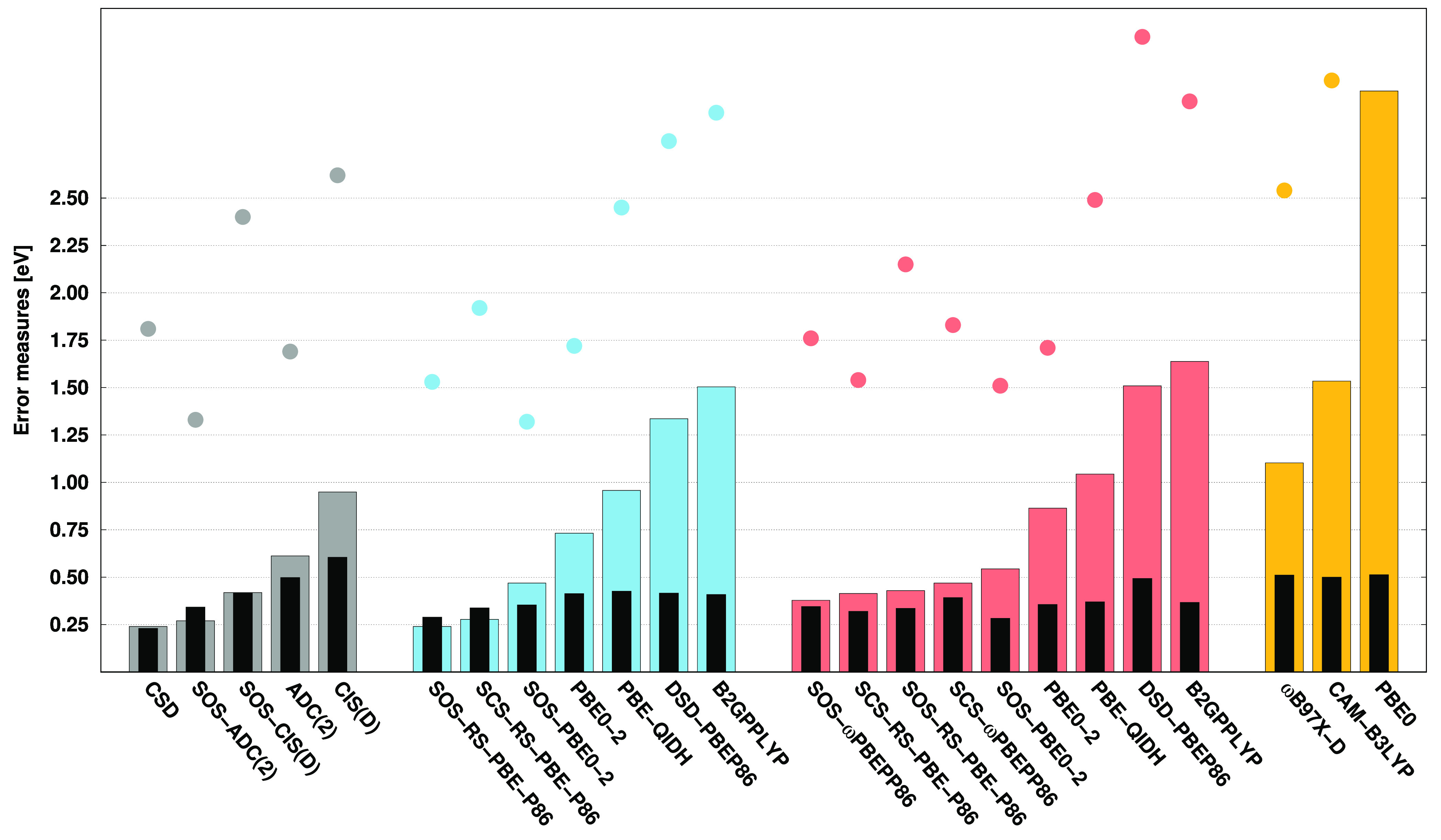
Error measures for the VIPs of the BO test set^47,123^ using the cc-pVTZ basis set with the corresponding auxiliary bases. See the caption of Figure 1 for further details.

Thereafter, the comprehensive BO benchmark set^47,123^ is assessed. The main error measures are collected in Figure 3. Upon inspection of the error bars, we can conclude that the best results are achieved by the CCSD and ADC(2)-based SOS-RS-PBE-P86 approaches, with MAEs of 0.24 eV. The performance of the SOS-ADC(2) and the SCS variant of the former functional is also outstanding, with error measures that do not exceed 0.30 eV. In addition, the CIS(D)-based variants of the SOS/SCS-RS-PBE-P86, SOS-PBE0-2, and SCS/SOS-ωPBEPP86 functionals can also be deemed reliable. Overall, the CIS(D)-based approaches are slightly outperformed by the ADC(2)-based ones. For this benchmark set, the ωB97X-D functional is not outstanding at all. The MAE is 1.10 eV, which is significantly higher in comparison with the best DH approaches. Similar rankings can be determined when considering SDs. Among the wave function-based methods, the most precise results are provided by CCSD, with an SD of 0.23 eV, while an SD of 0.34 eV is obtained for the SOS-ADC(2) approach. In this regard, the performance is more balanced for the DH functionals. The lowest SD, which is 0.29 eV, is attained by the ADC(2)-based SOS-RS-PBE-P86 method, while an SD of around 0.35 eV is achieved by the remaining best performers. Again, this error measure is noticeably higher for ωB97X-D, with an SD of 0.51 eV. Upon examination of the MAXs, the lowest value of 1.32 eV is obtained for the ADC(2)-based SOS-PBE0-2 approach, while a MAX of 1.33 eV is attained by SOS-ADC(2). The ADC(2)-based SOS-RS-PBE-P86 is also outstanding, with an error of 1.53 eV, but most of the DH functionals are reliable, as the MAXs do not exceed 2.00 eV, whereas it is 1.81 for CCSD. A somewhat higher value, precisely 2.64 eV, is obtained for ωB97X-D.

### 4.3 Nucleobases

**Figure 4:**
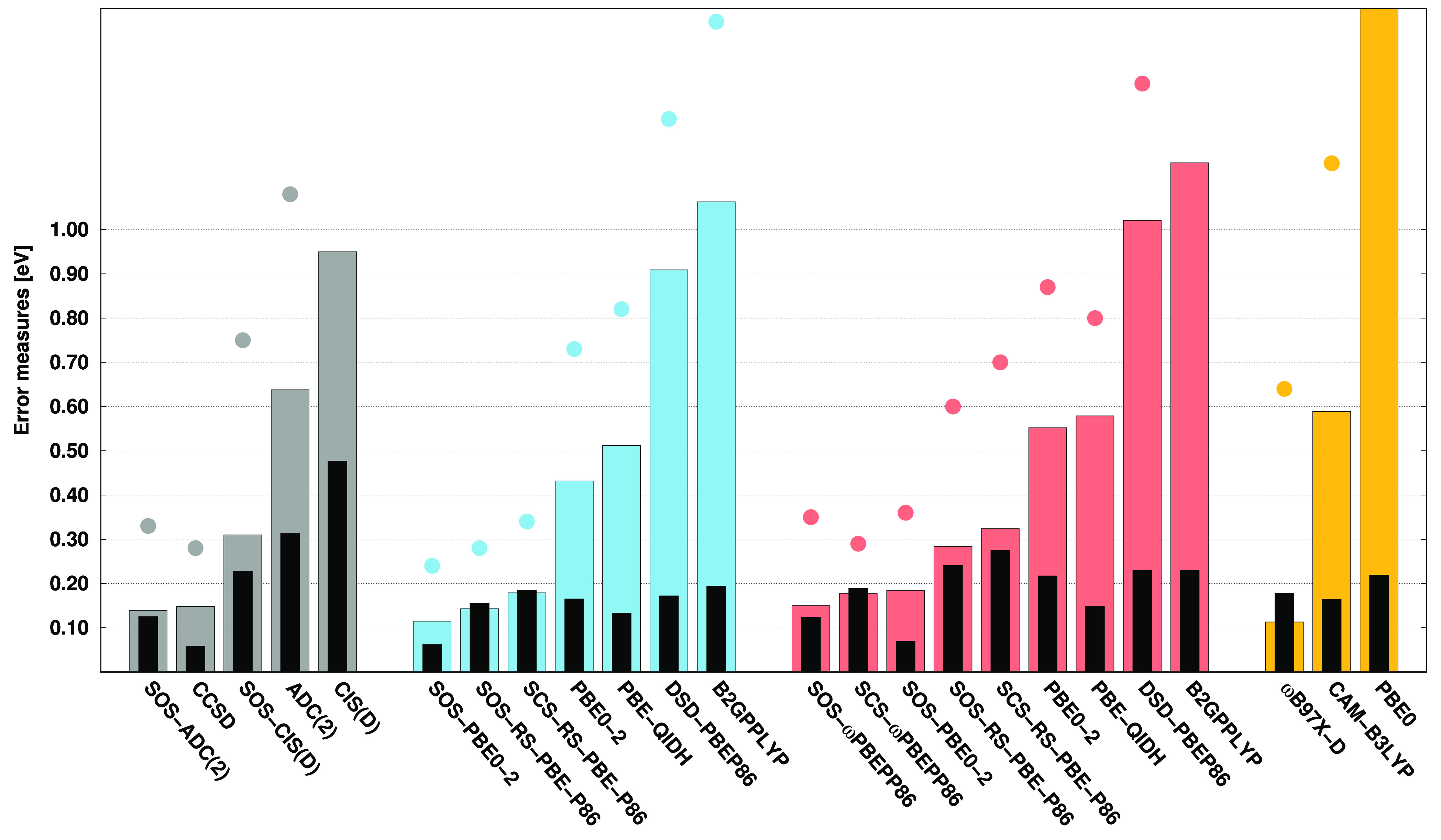
Error measures for the VIPs of the nucleobases^124,125^ using the cc-pVDZ basis set with the corresponding auxiliary bases. See the caption of Figure 1 for further details.

Finally, the performances for the nucleobases^124,125^ are discussed. The error measures corresponding to the VIPs are presented in Figure 4. On the basis of the numerical results, surprisingly, the ADC(2)-based SOS-PBE0-2 and ωB97X-D are the best performers, with MAEs of 0.11 eV. Similarly, outstanding accuracy is achieved by the SOS-ADC(2) and ADC(2)-based SOS-RS-PBE-P86 methods, while the CCSD and SOS-ωPBEPP86 approaches also seem to be reliable. In these cases, the MAEs are below 0.15 eV. Comparing the ADC(2)- and CIS(D)-based functionals, again, the former ones outperform the latter ones. When examining the SDs, similar conclusions can be drawn. That is, excellent precision is observed for the SOS-PBE0-2 functionals, with deviations of around 0.06 eV, and CCSD also achieves a similar result. In this regard, the ADC(2)-based methods provide more balanced values. The SD is approximately 0.15 eV for the remaining DH functionals, which is highly acceptable, while a bit larger values are obtained for the ωB97X-D and the CIS(D)-based approaches, except for SOS-ωPBEPP86. The MAXs are also well-balanced for the best DHs, ranging between 0.24 and 0.36 eV, while it is 0.64 eV for ωB97X-D.

**Figure 5:**
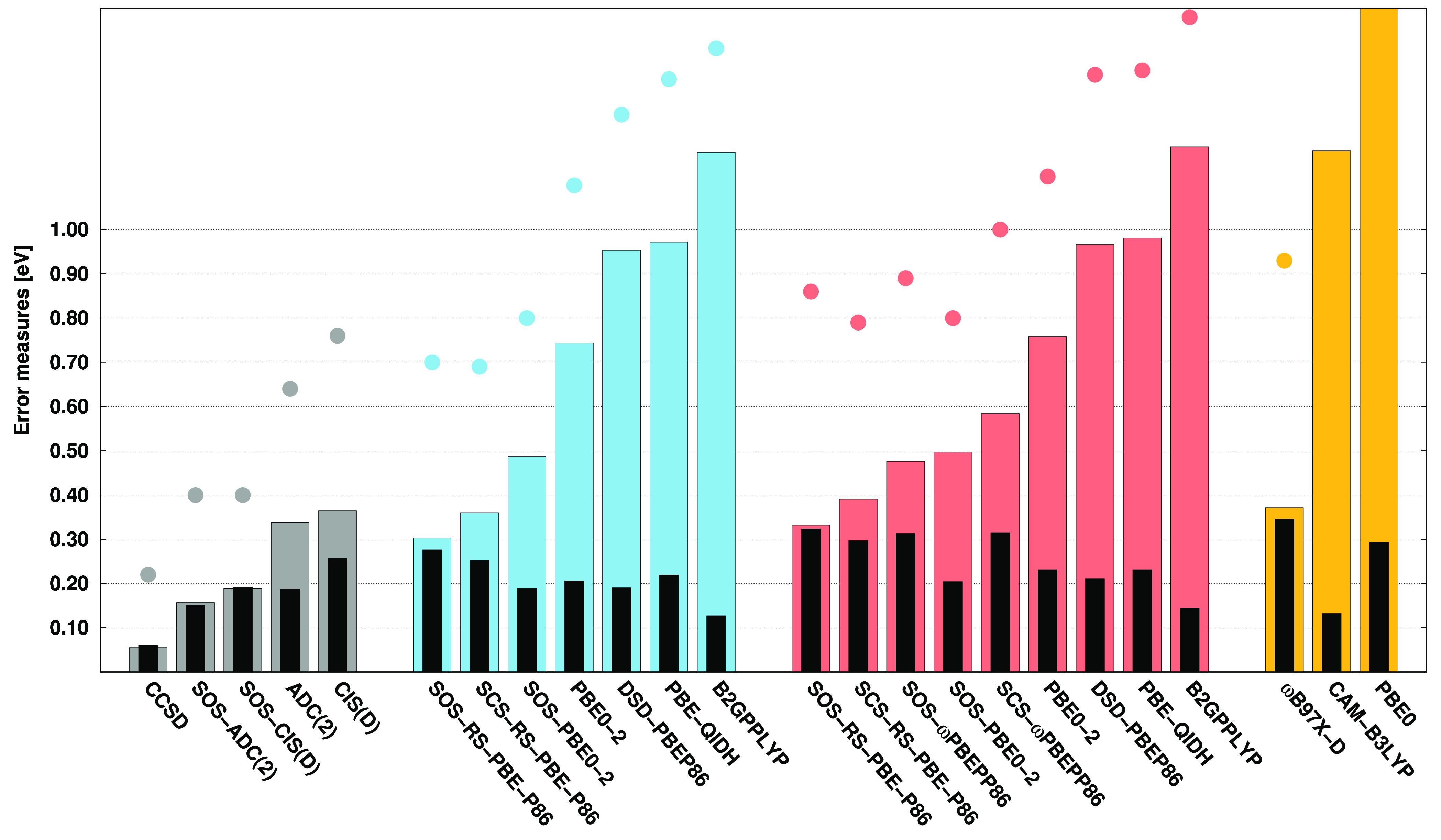
Error measures for the VEAs of the nucleobases^124,125^ using the cc-pVDZ basis set with the corresponding auxiliary bases. See the caption of Figure 1 for further details.

The results for the electron-attached states of the nucleobases are collected in Figure 5. As can be seen, again, CCSD and the SOS variants of the wave function-based methods outperform the DH functionals. However, the differences are somewhat less pronounced than those obtained for the acceptor test set. That is, the lowest MAE, 0.06 eV, is attained by CCSD, while the SOS-ADC(2) and SOS-CIS(D) approaches achieve MAEs of 0.16 and 0.19 eV, respectively. Among the DH methods, the most accurate approach is the ADC(2)-based SOS-RS-PBE-P86, with a MAE of 0.30 eV. In addition, the CIS(D)-based counterpart is also superior in its class. The SCS variant of the former functionals, as well as the SOS-PBE0-2 and SCS/SOS-ωPBEPP86 methods provide acceptable performance. In these cases, the MAEs do not exceed 0.50 eV. For the remaining DH functionals, significantly higher errors are obtained, while the MAE is 0.37 eV for the ωB97X-D method. Upon examining the SDs, an interesting behavior can be observed. Regarding the DH approaches, the lowest deviations are yielded by the methods that are the most inaccurate. For instance, B2GPPLYP has an SD of 0.13 eV, while this value is larger even for SOS-ADC(2). The deviations are around 0.26 eV for the most accurate ADC(2)-based approaches, while they are approximately 0.30 eV for the CIS(D)-based counterparts. A similar precision is achieved by ωB97X-D, with an SD of 0.35 eV. The lowest MAX, being 0.22 eV, is reached by CCSD, while SOS-ADC(2) and SOS-CIS(D) are also outstanding in this regard. The maximum errors are 0.40 eV for the SOS second-order wave function methods. The performance of the best DH functionals is well-balanced within each class. The MAXs are around 0.70 eV for the ADC(2)-based approaches, while they are roughly 0.85 eV for the CIS(D)-based ones. For the hybrid functionals, the lowest MAX, precisely 0.93 eV, is attained by ωB97X-D.

**Figure 6:**
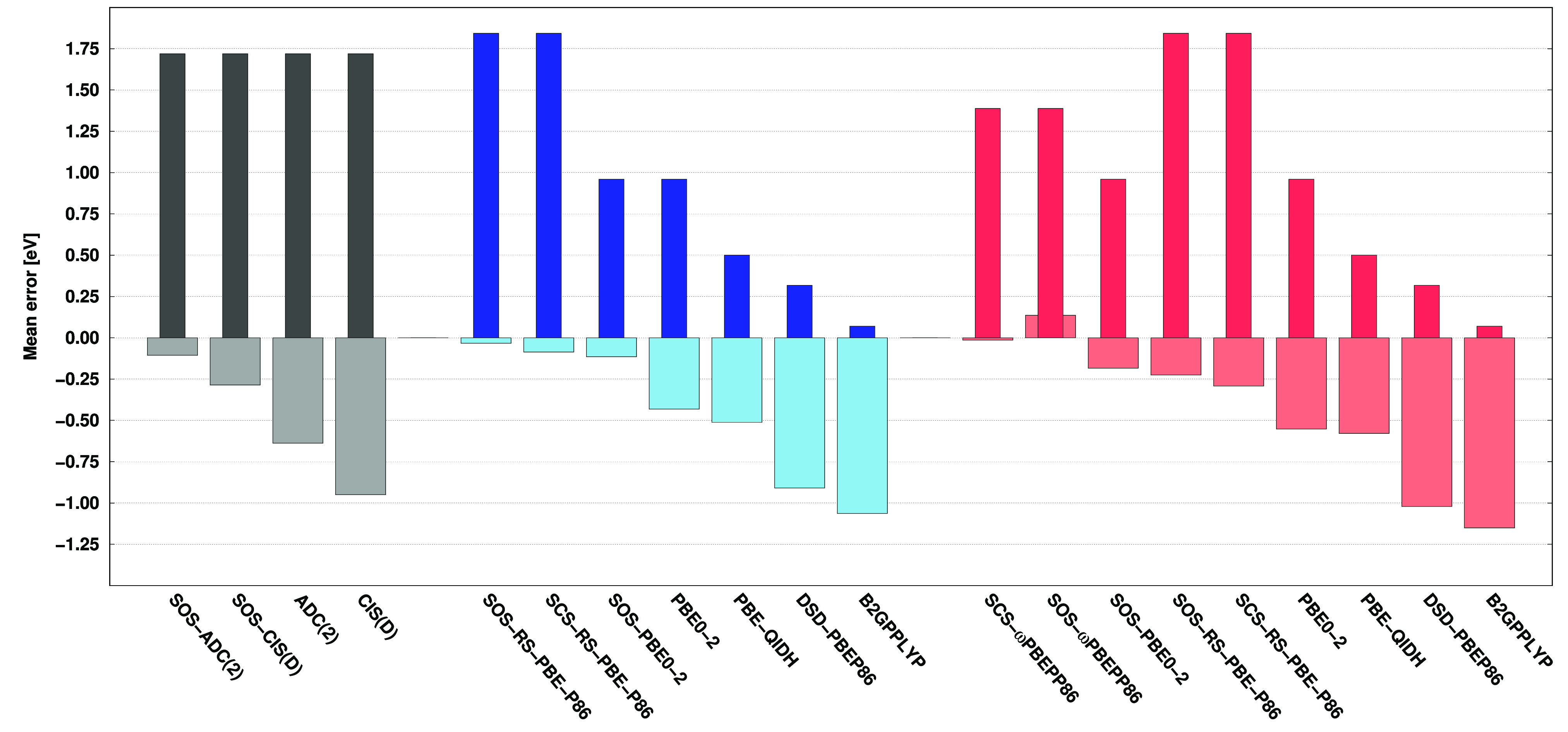
Mean errors for the VIPs of the nucleobases using the aug-cc-pVDZ basis sets with the corresponding auxiliary bases. Errors calculated from the orbital energies (final DH results) are visualized by the darker (lighter) bars. The wave function (first group), ADC(2)-based DH (second group), and CIS(D)-based DH (third group) methods are presented in gray, blue, and red, respectively.

For this benchmark set, the effect of the second-order correction is also assessed. This study is also intriguing due to the fact that orbital energies can be interpreted as VIPs and VEAs within Koopmans’ theorem. However, it is important to keep in mind that these values only offer a simplified approximation of the corresponding quantities as the relaxation effects are neglected. For the VIPs, the mean errors are collected in Figure 6, while the corresponding chart for the VEAs can be found in the SI. Considering the negative values of occupied orbital energies, it can generally be concluded that VIPs are consistently overestimated. This is also true for the wave function-based methods. The second-order correction, to some extent, systematically reduces the VIP values. As can be seen, the methods that are least accurate inspecting the orbital energies, such as SOS-ADC(2), ADC(2)-based SOS-RS-PBE-P86, and SCS-ωPBEPP86, actually provide the most accurate VIPs in terms of the final result. In contrast, for methods where VIPs can be precisely described by the negative of orbital energies, such as DSD-PBEP86 and B2GPPLYP, the second-order correction significantly reduces the corresponding values, leading to inaccurate results for the DH calculations. Similar findings can be reported if VEAs are inspected; however, the effects are opposite to what was observed for VIPs. For the most precise methods, virtual orbital energies significantly underestimate VEAs, and the second-order correction systematically increases their values. For the (SOS-)PBE0-2 and DSD-PBEP86 methods, VEAs can be precisely described by the virtual orbital energies, while for the least accurate methods, such as B2GPPLYP, the orbital energies are already overestimated.

### 4.4 Overall Performance

It is hard to characterize the performance of the methods with a single measure. Recently, a very simple procedure was suggested by Casanova-Páez and Goerigk.^77^ In their original paper, the MAEs obtained for the benchmark sets were simply averaged. In this study, we use the same measure separately for VIPs and VEAs. From a practical point of view, the computational cost of the approaches is also important. In order to help with the selection of methods worth applying, the overall performance of the best approaches and the rate-determining steps of the calculations are collected in Table 2. Inspecting the results, we can conclude that the most reliable method is the most expensive CCSD, while the SOS-ADC(2) approach is also outstanding. Among the functionals, the most robust performance is attained by the ADC(2)-based SOS-RS-PBE-P86 method. For ionization potentials, it outperforms SOS-ADC(2), and almost CCSD quality can be achieved. This method also provides the lowest overall MAE for VEAs; however, the accuracy is far below that obtained for CCSD or SOS-ADC(2). As a cost-effective alternative, SOS-ωPBEPP86 is also recommended for VIPs, but its performance is even less reliable for electron-attached states.

**Table 2:** Averaged MAEs for VIPs and VEAs, as Well as the Number of Independent Parameters and the Scaling of the Rate-Determining Steps on Top of the KS/HF Solution for the Best Performers

		MAE			Scaling	
Class	Method	VIP	VEA	Number of parameters	Ground state	VIP/VEA
wave function-based	CCSD	0.153	0.051	–	iterative *N*^6^	iterative *N*^5^
	SOS-ADC(2)	0.211	0.135	1	perturbative *N*^4^	iterative *N*^4^
	SOS-CIS(D)	0.325	0.146	1	perturbative *N*^4^	perturbative *N*^4^
	ADC(2)	0.585	0.431	–	perturbative *N*^5^	iterative *N*^4^
	CIS(D)	0.834	0.492	–	perturbative *N*^4^	perturbative *N*^4^
ADC(2)-based DH	SCS-RS-PBE-P86	0.212	0.458	4	perturbative *N*^5^	iterative *N*^4^
	SOS-RS-PBE-P86	0.182	0.354	3	perturbative *N*^4^	iterative *N*^4^
	SOS-PBE0-2	0.300	0.506	3	perturbative *N*^4^	iterative *N*^4^
CIS(D)-based DH	SCS-RS-PBE-P86	0.328	0.511	4	perturbative *N*^4^	perturbative *N*^4^
	SOS-RS-PBE-P86	0.306	0.396	3	perturbative *N*^4^	perturbative *N*^4^
	SCS-ωPBEPP86	0.256	0.683	7	perturbative *N*^4^	perturbative *N*^4^
	SOS-ωPBEPP86	0.226	0.552	5	perturbative *N*^4^	perturbative *N*^4^
	SOS-PBE0-2	0.352	0.518	3	perturbative *N*^4^	perturbative *N*^4^
LC hybrid	ωB97X-D	0.457	0.406	18	–	–
	CAM-B3LYP	0.924	1.121	3	–	–

## 5 Conclusions

In this study, double-hybrid time-dependent density functional theory has been extended to directly calculate vertical ionization potentials and electron affinities. The approach has been presented for the genuine variant of the DH TDDFT theory,^71^ where the second-order correction is calculated perturbatively relying on the CIS(D) method. In addition, our ADC(2)-based DH variant^82^ has also been considered, where the double excitations are treated iteratively. It has been demonstrated that the costs of the VIP and VEA calculations with the CIS(D)-based DHs are just comparable to one KS iteration step per state, while for the ADC(2)-based ones, the most demanding step is proportional to *N*^5^. However, it has to be carried out only once, regardless of the number of tackled states, and the iterative procedure still scales as *N*^4^. For a detailed comparison, state-of-the-art RS-DH and LC-DH methods, including spin-scaling techniques, were selected, as well as robust and popular hybrid and global DH approaches were also included. To assess the performance of the functionals, comprehensive benchmark calculations were carried out on the best available test sets. A total of 224 electron-detached and 54 electron-attached states were examined, with references provided by higher-order coupled-cluster calculations that include triple excitations.

The findings were analyzed from two main perspectives. First, the robustness of the ADC(2)-based ansatz was inspected in contrast to the CIS(D)-based approach. In this regard, the advantages of the more advanced formalism are clearly demonstrated. Second, the impact of the DH approach was assessed in comparison with the corresponding wave function-based methods. The excellent performance of SOS-ADC(2) had already been revealed by Tajti and co-workers.^124^ Accordingly, the efficiency of the approaches is compared to this starting point. Our numerical results show that, among the functionals, the most robust performance is attained by the ADC(2)-based SOS-RS-PBE-P86 approach. This method consistently outperforms SOS-ADC(2) for ionization potentials; furthermore, CCSD quality can also be reached. Unfortunately, the results are not so encouraging for electron affinities. Even though the best performance is achieved by the above functional, the accuracy is far below that obtained for SOS-ADC(2). For ionization potentials, the CIS(D)-based SOS-ωPBEPP86 is also recommended, while SOS-PBE0-2 is identified as the most reliable global DH functional. The importance of spin-scaling techniques was confirmed by this study as well, though the performance of DSD-PBEP86 is found to be somewhat disappointing. In addition, surprisingly good results were revealed for ωB97X-D, where the VIPs/VEAs are associated with the corresponding Kohn–Sham orbital energies within the present formalism. Nevertheless, ωB97X-D does not outperform the DHs concerning robustness.

## Supporting Information Available

Computed vertical ionization potentials and electron affinities (XLSX)
